# Crosstalk between Light- and Temperature-Mediated Processes under Cold and Heat Stress Conditions in Plants

**DOI:** 10.3390/ijms22168602

**Published:** 2021-08-10

**Authors:** Tibor Janda, Sylva Prerostová, Radomíra Vanková, Éva Darkó

**Affiliations:** 1 Centre for Agricultural Research, Department of Plant Physiology and Metabolomics, Agricultural Institute, ELKH, H-2462 Martonvásár, Hungary; darko.eva@atk.hu; 2 Laboratory of Hormonal Regulations in Plants, Institute of Experimental Botany, Czech Academy of Sciences, 16502 Prague, Czech Republic; prerostova@ueb.cas.cz (S.P.); Vankova@ueb.cas.cz (R.V.)

**Keywords:** acclimation, climate change, cold, heat, light, photosynthesis, phytochromes, signalling, temperature

## Abstract

Extreme temperatures are among the most important stressors limiting plant growth and development. Results indicate that light substantially influences the acclimation processes to both low and high temperatures, and it may affect the level of stress injury. The interaction between light and temperature in the regulation of stress acclimation mechanisms is complex, and both light intensity and spectral composition play an important role. Higher light intensities may lead to overexcitation of the photosynthetic electron transport chain; while different wavelengths may act through different photoreceptors. These may induce various stress signalling processes, leading to regulation of stomatal movement, antioxidant and osmoregulation capacities, hormonal actions, and other stress-related pathways. In recent years, we have significantly expanded our knowledge in both light and temperature sensing and signalling. The present review provides a synthesis of results for understanding how light influences the acclimation of plants to extreme low or high temperatures, including the sensing mechanisms and molecular crosstalk processes.

## 1. Introduction

Climate change may exacerbate the frequency and severity of a number of stresses. It leads to increasing air CO_2_ level and global temperature with extreme temperature waves, and it may also cause dramatic changes in precipitation, which altogether may have multiple effects on crop production. These kinds of stresses alone, or in combination, modify plant growth and development, and, in most cases, lead to significant economic losses worldwide. For example, heat stress was estimated to reduce the yield of wheat, which is one of the most important crop species, by approximately 15% [1]. However, the net effect of climate change on crop yield depends on the interactions among different environmental factors.

Although the yield of many crop plants showed increasing trends in the last decades, the yields of key crops have reached a plateau in recent years. Since more than 10% of the total world population still suffers from undernourishment, continued fast increases in crop yields are required to feed the growing world population. Due to the agronomic importance of abiotic stressors, plant breeders and crop producers are actively seeking stress tolerance characteristics and a sustainability of yield under stress conditions. In order to develop crop plants tolerating environmental changes with as little damage as possible, understanding of plant defence mechanisms, regulatory processes, and genes responsible for stress tolerance is essential.

Extreme temperatures are among the most important stressors limiting plant growth and development. Acclimation to suboptimal temperatures causes mild stress, but it also induces several protective mechanisms, which may help to survive the extreme environmental deviation. For instance, growing under slightly elevated temperatures may lead to an increased heat tolerance, manifested in higher survival rate and even higher crop yield. This is called acquired thermal tolerance, established via heat acclimation or heat priming processes. It has also been demonstrated that heat priming at the pre-anthesis stage reduced the damage caused by post-anthesis heat stress in wheat leaves [2]. 

On the other side, growing at low, but above zero temperatures may increase freezing tolerance in various plant species. This is called cold hardening, and, even in the case of plant genotypes with the highest levels of genetically determined frost tolerance, it is important that plants are exposed to low, but above-freezing temperatures before the onset of extreme frost [3]. However, when autumn temperatures remain high, plants cannot always be prepared for the winter period, and, in spite of global warming, they may suffer more severe freezing injury. The same is true for crop plants of tropical or subtropical origin, such as maize, whose growing regions have been shifted to the north. A sudden temperature drop in spring may cause severe chilling injury. Recent results also showed that both moderate water deficiency and light intensity may also have an influence on cold hardening, indicating a crosstalk mechanism between different stress responses [4]. However, the imitation of mild heat or cold acclimation periods is complicated in real field conditions.

Interaction of different kinds of environmental factors, such as the above-mentioned light or water deficit, also affect the temperature acclimation processes. In the last years, the light-dependent acclimation processes started to be in focus for a couple of reasons. One reason is the growing amount of evidence indicating the role of light intensity and spectral composition in the development of stress tolerance. This issue will become even more important in the future prospect of changing global climate. The second reason is that the spread of light-emitting diode (LED) technology in greenhouse-based crop production has raised new questions about light intensity and quality when examining the role of light in the development of the most efficient cultivation technologies.

The interaction between light and temperature in the regulation of stress acclimation mechanisms is complex. It involves various processes, including regulation of stomatal movement, nitrogen metabolism, antioxidant and osmoregulation capacities, and signal transduction in stress-related pathways. Since this question is both of theoretical and practical importance, in the present review, we focused on the light- and temperature-sensing mechanisms and interactions between the acclimation to high or low temperatures and the light-mediated processes. 

## 2. Light Perception

Light is one of the most important environmental factors for plants, which can be sensed by special pigments and photoreceptors. Moreover, a significant proportion of the compounds in plants are also able to absorb light in a certain wavelength range; however, the changes in molecules caused by light absorption do not always induce physiological processes. The most important example of light absorption relates to photosynthesis. It has been suggested that, in addition to its well-known role in capture and transduction of light energy, the photosynthetic apparatus might also act as an environmental sensor [5]. On the other hand, several other light receptors are also known and play a significant role in the regulation of plant growth and development, and they may also influence the adaptation to different environmental factors. 

Light signalling is often mediated by discrete protein photoreceptors. Phytochromes (Phys), cryptochromes (Crys), phototropins (Phots), zeitlupes (ZTL, FKF1, and LKP2), and UV Resistance Locus 8 (UVR8) are the best-known photoreceptors in plants, which can be characterised with different wavelength specificities. Phys absorb mainly in the red and far-red region, Crys, Phots, and zeitlupes in the ultraviolet (UV)-A/blue ranges, and UVR8 operates through UV-B light (280–315 nm) [6]. Crys seem to also absorb in the green light region. After absorption of the specific light wavelengths, they may undergo a conformational shift, leading to a change in their activation and/or function [7].

Phys are the most widely known photoreceptors. According to their protein structure, several isoforms (such as PhyA-E in Arabidopsis) exist, but they all use phytochromobilin as the light-absorbing chromophore. Phy composition may also differ in the different plant species [8]. PhyA and PhyB are the most abundant phytochromes in dark-grown and de-etiolated seedlings, respectively. PhyB is present in two alternative isoforms, the inactive Pr, which absorbs maximally at the red region, and the active Pfr, with a maximum absorbance in the far-red region. Exposure of Pr to red light induces a change to Pfr form, and, vice versa, far-red illumination accelerates the conversion of Pfr to Pr. Pfr form may also spontaneously relax into Pr, which is called dark or thermal reversion. The inactive PhyB in Pr state is located in the cytoplasm. When PhyB is in Pfr, it may enter the nucleus. Phys can interact with phytochrome-interacting factors (PIFs). These transcriptional factors are characterised with basic helix–loop–helix (bHLH) DNA-binding domain. Interaction between Pfr and PIF4 has recently been reviewed [9]. In the PhyB prevalence in cytoplasm, PIF4 may bind to the regulatory region of target genes and regulate their expression through its bHLH domain. When PhyB in Pfr form is abundant in the nucleus, PIF4 binds to active PhyB and is sequestrated from target genes [10]. Besides deactivation, PIF4 may also be degraded by 26 proteasomes through ubiquitination. The abundance of PIF4 is partly controlled by two BLADE-ON-PETIOLE proteins (BOP1 and BOP2). BOP proteins have been shown as important regulators of the growth and development of lateral organs, including, among others, control of axillary bud growth or nodule formation in legumes [11]. BOP proteins physically interact with both PIF4 and CULLIN3A (subunit of E3 ligase in CRL3 complex). CULLIN3–BOP2 complex may ubiquitinate PIF4, targeting it to proteasome degradation [12,13]. 

Crys are blue-light receptors presented from bacteria to humans. In plants they modulate responses from germination to fruiting. In Arabidopsis, three Crys (Cry1, Cry2, and Cry3) were identified. Cry1 and Cry2 are located predominantly in the nucleus, while Cry3 is in chloroplasts and mitochondria. Cry3 repairs UV-damaged DNA in a light-dependent manner [14]. 

Phots regulate key physiological responses, such as phototropism of shoots and roots, stomatal opening, leaf expansion, and seedling elongation [15]. Recent study indicates that they are repeatedly duplicated and diverged, and their individual forms mediate different light responses [16]. The zeitlupe proteins are light–oxygen–voltage (LOV)-domain-based blue-light photoreceptors. They control protein degradation by ubiquitination, and they may play a role in the regulation of the circadian clock and photoperiodic flowering [17]. UVR8 protein is localized in both the nucleus and cytoplasm, and it also participates in the regulation of different plant growth and developmental processes. UVR8 does not have a prosthetic chromophore, but it uses tryptophan residues as chromophores. In the nucleus, it may bind to several UV-B responsive genes, including the Elongated Hypocotyl 5 (HY5) promoter region [18]. The present summarised results suggest that plants have evolved complex light-sensing systems, which also take part in the development of temperature acclimation processes. However, the links between the light-sensing and activation mechanisms are still poorly understood.

## 3. Temperature Sensing

While several light receptors have been described and characterised, the primary temperature sensing processes are still poorly understood. The molecular mechanism of thermosensing in plants has only been characterised in Arabidopsis in the last decade. It was shown that a histone variant H2A.Z plays an essential role in perceiving the ambient temperatures. Plants which are deficient in incorporating H2A.Z into nucleosomes show a phenotype of plants growing at elevated temperatures. Furthermore, nucleosomes containing H2A.Z may respond to temperature independently of transcription through DNA-nucleosome fluctuations [19]. Higher temperatures lead to eviction of H2A.Z from nucleosomes, enabling heat shock transcription factor HSF1 to begin fast transcription of stress-responsive genes [20].

PhyB has also been suggested as a potential temperature sensor, so it may sense both light and temperature [21,22]. Not only far-red light, but high temperatures may also increase the speed of conversion of activated PhyB (Pfr) into the inactive form (Pr), resulting in increased stabilisation of the transcription factor PIF4 [23]. As a consequence, the PIF4 protein was found to be more stable under relatively high temperature conditions, especially in the nuclei [24]. Conversely, PIF4 can be degraded not only under red light, but at low temperatures too [25]. 

Although the fact that Phys are involved in the temperature sensing was suggested a relatively long time ago, the exact molecular mechanisms have only been elucidated in recent years, at least partly, by Meng Chen’s group [26,27]. One of their recent studies, using a missense allele hmr-22 of Arabidopsis plants, revealed a novel PhyB-mediated temperature-signalling mechanism, in which the transcriptional activator HEMERA (HMR) directly interacts with PIF4, facilitating the activation of thermoresponsive PIF4-target genes, and also the PIF4 protein accumulation [26]. Inside the nucleus, Phys can be combined into units called photobodies [28]. Although, not only shade may antagonise PhyB signalling by triggering PhyB disassembly from photobodies, but warm temperatures could work similarly; however, elevated temperatures elicit different photobody dynamics than shade [27]. The thermostability of photobodies relies on the photosensory module of PhyB. Increases in temperature may reduce photobody number by stimulating PhyB disassembly from selective thermo-unstable photobodies. It also means that different temperature ranges may selectively reduce specific photobodies. Furthermore, the response may differ in individual organs, as was observed in hypocotyl and cotyledon. These results suggest that, since dynamic assembly/disassembly of individual photobodies may have distinct thermostabilities, individual photobodies could be sensors for specific temperature ranges [27].

Recently, it was also demonstrated that the lifetime of the photoactivated blue-light photoreceptor phototropin is temperature dependent, indicating that phototropin can also act as a potential thermosensor. The photoactivated phototropin is thermosensitive and a higher temperature deactivates it faster than cold. As a consequence of the long-term active phototropins, chloroplasts move to sides under cold treatment, and they try to avoid blue light under cold conditions [29]. 

There are also other ways plant cells may sense changing temperatures. High temperatures may aggregate proteins. This can be sensed by heat shock proteins (HSPs), leading to the release of HSFs. They may bind to heat shock elements, and induce transcription of genes related to heat responses. However, most of the HSPs, which may keep protein homeostasis through their chaperone activities, are also induced by other stressors, so not all of them may serve as a specific temperature sensor. Furthermore, light-responsive cis-acting regulatory elements were also detected in wheat HSPs, indicating a close relationship between the light- and temperature-regulated processes [30]. 

A product of the chilling-tolerant divergence 1 (COLD1) gene has also been proposed as a potential temperature-sensing component [23]. COLD1 encodes a transmembrane protein that interacts with a G-protein, which results in enhanced GTPase activity and faster Ca^2+^ influx into the cell upon exposure to low temperatures. It has been shown that a single nucleotide polymorphism in the COLD1 gene is responsible for the difference in cold tolerance between indica and japonica rice plants [31]. 

Roots may also have independent thermo-sensing mechanisms [32]. Earlier studies of intact root systems in Arabidopsis demonstrated that the Ca^2+^ influx mediated by a special Ca^2+^ channel depends mainly on the cooling rate, and not on the absolute temperature. This means that Ca^2+^ influx rate may turn back to a normal level when the low temperature becomes constant. However, the responsiveness of the temperature-sensing system to lower temperatures acts on another mechanism, and it depends on the absolute temperature [33]. While the heat-sensing mechanisms are at least partly characterised in Arabidopsis, they are much less known in crop plants, where the primary cold sensors have not been clarified yet [34].

Changes in temperature directly affect all metabolic pathways, including modification of membrane fluidity. In long-term, both animal and plant cells may respond to changing temperatures with alteration in membrane composition. Changes in triacylglycerol contents and degree of saturation level of fatty acids have been associated with plant cold tolerance. In wheat chromosome substitutional lines, the decrease in the trans-∆3-hexadecenoic acid content correlated with frost tolerance [35]. However, although adjustment of membrane fluidity may mediate the acclimation processes to extreme temperatures, it is probably not the primary temperature-sensing mechanism [23].

Results focusing on the primary temperature-sensing processes already suggest that temperature- and light-sensing mechanisms may at least partly overlap. One of the best examples is the similar responses of PhyB to changes in light spectrum and elevation in temperature. However, detailed studies also show differences in these processes. Better understanding of the temperature-sensing processes would also help to better understand the acclimation mechanisms to low or high temperatures in plants.

## 4. Light Signalling Leading to Cold Tolerance

### 4.1. Light Intensity as a Signal

**Figure 1:**
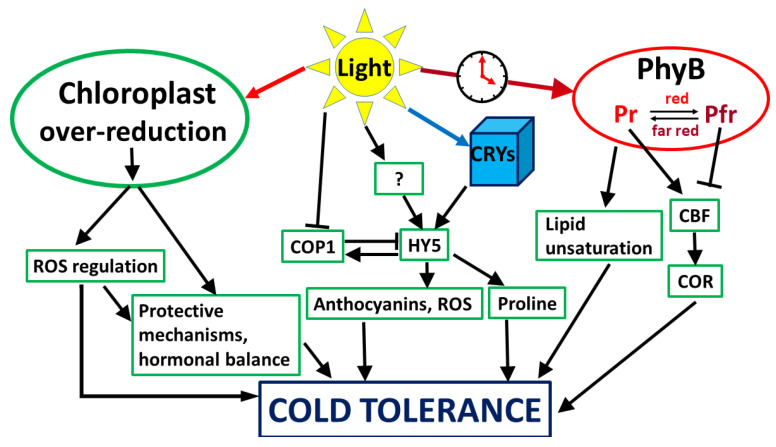
Possible main factors playing a role in light–low-temperature interactions in plants. There are two main routes for how light during the acclimation period may improve cold tolerance of plants. One is related to the photosynthetic processes, where the excitation pressure of the electron transport chain may induce protective mechanisms. In this case, light intensity may also determine the level of cold tolerance. The other way is determined by light spectrum, and it is related to photoreceptors, such as PhyB and Crys, which may also mediate stress-related processes. For more details, see text.

The molecular, physiological, and genetic mechanisms underlying the elevation of freezing tolerance have been intensively studied for several decades. In spite of the fact that many details are still poorly understood, the main processes have been well characterised [36,37]. Growing plants at low, hardening temperature itself is not sufficient for developing a high level of frost tolerance. The acquisition of efficient cold hardiness requires both low temperature and light (Figure 1). Hardening in the dark, or under low light conditions, is much less effective than at normal or high lights [3,38,39]. The fact that growing under high light conditions without exposure to cold may also induce partial freezing tolerance led to the original view that the main signal for the development of light-induced freezing tolerance may arise from the overexcitation of the photosynthetic electron transport chain, the excitation pressure, and the redox state of plastoquinone [38]. The direct role of chloroplasts in cold acclimation has been demonstrated using barley mutants with impaired chloroplast development, which also lost the ability to develop freezing tolerance [40]. Using microarray analysis, impairment of chloroplast development reduced the induction of almost 90% of the cold-inducible genes, and the majority of them were not regulated by cold at all [41]. Interestingly, C-repeat binding factor genes (CBFs), which play a key role in the development of cold tolerance, were not influenced by the mutation, indicating that they are chloroplast-independent. In contrast, light enhanced the induction of CBF genes in Arabidopsis plants [42] and in cereals [43]. It must be emphasized that induction of the CBF genes depends not only on the light intensity, but also on the light spectrum (see next subchapter).

Further works have characterised several light-regulated cold-hardening processes. For example, genes in Arabidopsis that were more upregulated at low temperatures in the light than in the dark include various stress-related genes, among others, XERO2/LTI30 and RAB18 encoding dehydrin proteins, the Cold Regulated 78 (COR78) gene, Early Response to Dehydration (ERD3 and ERD10), or the gene coding stress-induced protein KIN1, which functions as an antifreeze protein [44]. Transcription of KIN1 is also induced by abscisic acid (ABA), dehydration, or osmoticum. Exposure to low, hardening temperature may also downregulate the expression of certain stress-related genes. Cold tolerance in plants can also be negatively regulated by certain proteins, for example, by Eskimo (ESK1), which also plays a role in the regulation of water balance [45]. Furthermore, galactinol synthase genes have also been upregulated, which might play a role not only in cold adaptation, but also in acclimation to high temperatures in cereals [46].

The most important practical significance of enhanced frost resistance is associated with the winter hardiness of winter cereals. Microarray-based transcriptomic analyses in winter and spring wheat varieties have shown that expression level of a large number of genes was affected by light during the cold-hardening period, and these changes differed in winter and spring varieties too. Not surprisingly, photosynthesis-related genes were the ones most affected, which may also play a key role in the development of cold tolerance, because adjustment of the photosynthetic processes to lower temperature is an important component of cold adaptation processes. The induction or suppression of various genes by light may indicate either a crosstalk between the chloroplast-originating and photosynthesis-independent protective mechanisms or the involvement of different light sensors transducing light signals in order to improve freezing tolerance [47]. These results support that light- and temperature-mediated signalling processes function in a complex interaction, including chloroplast-related mechanisms, the expression level of stress-related genes, and the synthesis of various protective compounds, leading to high level of freezing tolerance [3,25].

As with the majority of environmental stress factors, low temperatures may also induce the production of reactive oxygen species (ROS). One of the possible mechanisms to reduce the level of cold injury is to maintain the ROS at optimum level [48]. ROS and reactive nitrogen species (RNS) may also induce signals, which modify gene regulation, hormonal levels, and enzyme activities [49,50,51,52,53]. The importance of ROS in cold acclimation can be deduced from the fact that genes induced by low temperature and hydrogen peroxide signalling may substantially overlap, as it was shown in cold-tolerant japonica rice plants [54]. At low temperature, the induction of genes playing a role in antioxidant protection also occurred in Arabidopsis only in the light, but not in the dark [44]. Similarly, under cold-hardening conditions, induction of antioxidant enzymes, such as glutathione reductase and ascorbate peroxidase, were detected in wheat only under normal growth light conditions, but not at low light [55]. Glutathione S-transferases or salicylic-acid-related processes are also mediated by light [56,57]. It seems that ROS production can be another connection point between the light- and cold-induced mechanisms. The critical process may be related to photoinhibition. This results from exposure of plants to light intensity higher than that which can be utilised in photosynthesis or eliminated in the regulating processes, reducing the capacity of the photosynthetic machinery (recently reviewed by [58]). Under stress conditions, when the energy utilisation is limited, photoinhibition may occur even under normal growth light conditions. This means that the production of ROS under stress conditions can be more pronounced.

Besides the antioxidant compounds, several stress-related protective compounds accumulated in wheat plants in a light-dependent manner during the low temperature hardening period, differing between the spring and winter varieties. These include, among others, polyamines, proline, salicylic acid, and related compounds [47,55,59]. These processes have already been reviewed [3]. The role of cytokinins during low temperature stress under different light conditions has recently been demonstrated in detailed physiological and proteome analyses using transgenic Arabidopsis lines with elevated or reduced cytokinin levels. Interestingly, light intensity significantly influenced the reactions to low temperatures not only in the leaves, but also in the shaded roots [60].

Although cold hardening is usually associated with the development of frost resistance of winter cereals, recent results show that species of subtropical origin (maize) can also be cold acclimated to some extent, requiring light signal as well [61]. The genes differentially expressed under variable light conditions were mainly involved in photosynthetic light and dark reactions, carboxylic acid metabolism, cellular amino acid, porphyrin or glutathione metabolic processes, and ribosome biogenesis. These results led to the conclusion that photoinhibition under cold conditions, connected with the overexcitation of the photosynthetic electron transport chain and reduction in the efficiency of photosystem 2, can be a “necessary evil” for cold acclimation processes in plants [61]. However, although more and more is known about the light dependence of cold acclimatisation processes, the direct link between the overexcitation of the photosynthetic electron transport chain by light and other temperature signalling processes is still missing.

### 4.2. Light Quality as a Signal 

Besides light quantity, light quality, i.e., the spectral composition, has also been shown to play a role in the cold responses, indicating that the photosynthetic machinery, as well as photoreceptors, are important in the development of cold tolerance (Figure 1). 

#### 4.2.1. Red Light

Early works on woody plants and recent studies on cereals also reveal that plant illumination with far-red or red lights during the cold-hardening period may significantly affect the cold tolerance [62,63,64]. The contribution of the photoreceptors to the development of freezing tolerance has been demonstrated by [65]. They showed that light was able to affect the expression of certain genes by either promoting photosynthesis or by activating Phy or the blue-light receptors in barley plants. Induction of co-ordinated expression of the COR genes is generally associated with increased hardening. The product of the COR14b gene is a chloroplast-localised protein. It can be efficiently accumulated during the cold hardening in the light, but not in the dark [66]. When etiolated plants were exposed for a short period to monochromatic red light before the cold-hardening period, COR14b was detected. However, far-red light reduced its efficiency, indicating that Phy played a role in COR14b accumulation. Similarly, blue light was also as effective as red light, suggesting Cry action [65]. This is in agreement with another study, in which the induction of COR15a, another cold-inducible, ABA-independent gene, was more pronounced by red than by far-red light in Arabidopsis. Furthermore, analysis of different Phy mutants suggested that PhyB is the primary photoreceptor, which mediates the white light signalling to the cold-induced gene expression through the C-repeat/dehydration-responsive (C/DRE) cis-acting element [42]. 

In contrast, light with low red to far-red ratio increased the CBF gene expression in Arabidopsis plants [67]. What can explain this contradiction? First of all, the light- and cold-dependent inductions of CBFs also depend on the circadian clock, and the peak of CBF expression may depend on both the red/far-red ratio and the ambient temperature [67,68]. The effects of the circadian clock and photoperiod on CBF-mediated cold tolerance have been summarized [69,70]. Furthermore, the light regulation of COR gene expression may also be driven by other enhancers than C/DRE elements. So, radiation with low red/far-red ratio light may elevate the expression of CBFs, and, consequently, the expression of COR genes at low temperatures [67]. PhyB and PhyD often act redundantly, and they may also synergistically regulate various physiological processes [71]. Since PhyB and PhyD mutants showed an increased expression of COR15a, it supports the view that these Phys may play as negative regulators of freezing tolerance. However, the picture is not so clear. PhyB mutant Arabidopsis plants were also characterised with reduced fatty acid desaturase (FAD) expression levels, leading to decreased polyunsaturated fatty acids [72]. This means that, while PhyB may reduce thermotolerance in this way, its effect on cold tolerance, due to a higher lipid unsaturation level, can be beneficial. 

Positive impacts of the low red/far-red ratio on freezing tolerance, determined as a reduced level of frost-induced membrane injury, have also been demonstrated in cereal species [73]. Furthermore, recent results also suggest that complementary, high far-red irradiation does not only affect the actual Phy activation state, but it may also have influence on the expression of PhyA and PhyB genes. However, the level and the duration of the increased expression rate are highly dependent on the plant genotype [43]. Based on the proposed model, under white light illumination, CBF14 is repressed by the active form of PhyB, and the expression of PhyA is inhibited by light and by PhyB. With decreased red/far-red ratio, PhyB is mainly inactive, and CBF14 and PhyA are released from the inhibition. Then, the active PhyA form may also enhance the expression of CBF14. PhyC has probably no significant impact on light-mediated freezing tolerance in cereals [43]. Among barley cold-inducible genes playing a role in freezing tolerance, the expression of HvCBF14 or HvCOR14b was highly responsive to light spectra, more than that of HvDHN5 gene encoding a dehydrin protein [73].

#### 4.2.2. Blue Light

The involvement of certain key regulators of light signalling, such as HY5, COP1, and the Z-box cis-regulatory element, which is activated by Crys through various interactions with other photoreceptors and signalling molecules, has also been shown in cold acclimation processes [14]. HY5 is a bZIP transcription factor, which serves as a positive regulator of cold hardening [74]. It is upregulated at low temperatures, and it mediates the induction of several cold-inducible genes through the Z-box elements. The transcript level of HY5 is regulated through a CBF- and ABA-independent pathway. The HY5 protein is post-translationally negatively regulated by the E3 ubiquitin ligase COP1, which interacts directly with HY5 in the nucleus and may repress the HY5-mediated signalling [75]. Light reduces the COP1 abundance in the nucleus by its exclusion, which enables HY5 to serve as an activator of light-responsive genes. HY5 may also positively regulate COP1 transcription, providing a negative feedback loop [76]. In Arabidopsis, HY5 may regulate approximately 10% of the cold-inducible genes, including genes encoding enzymes playing a role in anthocyanin biosynthesis, which may also protect plants from ROS accumulation under low temperature conditions [74]. Furthermore, recent results show that HY5 may also positively regulate the level of proline, an important osmoprotectant, as well as ROS scavenger and signalling compound [77]. HY5 also connects various hormonal signals, such as ABA, auxin, gibberellins, or cytokinins. Plant hormones, especially gibberellins and cytokinins, regulate HY5 through modulation of COP1: gibberellins inhibit, cytokinins activate HY5 [78]. In tomato plants, HY5 could directly activate transcription of genes encoding gibberellin-deactivating enzymes GA2ox and ABA biosynthetic enzyme 9-Cis-Epoxycarotenoid Dioxygenase 6 (SlNCED6). A PhyA-mediated SlHY5 accumulation resulted in an increased ABA/gibberellin ratio, which was accompanied by induction of cold response [79]. A recent detailed study also demonstrates the involvement of phytohormones in a complex crosstalk between light and temperature signals. Exposure of Arabidopsis plants to low, acclimating temperatures at optimal light intensity induced upregulation of the most physiologically active cytokinin trans-zeatin in the leaves and roots; the responses were mainly mediated by Crys. Under low light conditions, an increase in low active stress-response-related cytokinin cis-zeatin in apices was detected, and PhyA had the dominant role under these light conditions [80].

Taken together, all of these data suggest that light intensity plays a particularly important role in low-temperature acclimatisation. Several processes have become known that can be directly or indirectly influenced by the intensity of light, and several of these can be related to the development of cold tolerance. However, the direct link between the light intensity and signal transmission systems is not yet known. In addition, different photoreceptors are also able to affect cold tolerance in plants.

## 5. Influence of Light on Acclimation to Elevated Temperatures

Due to global warming, a better understanding of the heat acclimation processes of plants becomes increasingly important, as this knowledge may contribute to improving heat tolerance of plants. The acclimation ability of plants to extreme temperatures strongly depends on the genotypes and may vary from species to species. However, analysis of various plant species showed much less variation in heat than in cold tolerance [81]. High temperatures modulate various molecular and physiological processes in living organisms. For example, in crop plants, the decrease in yield caused by high temperatures is partly due to reduced photosynthetic CO_2_ assimilation capacity and increased mitochondrial respiration [82]. 

Growing at elevated, but non-lethal, temperatures may improve the heat tolerance of plants via, among others, increasing the thermotolerance of the photosynthetic apparatus [83]. After returning to optimum temperatures, the primed state can be maintained for a few days, which is also referred to as heat stress memory [84]. Several molecular mechanisms are involved in the heat acclimation, including signalling processes modifying the expression level of stress-related, especially photosynthesis-responsive, or antioxidant-enzymes-related genes, the synthesis of osmoprotectants, and HSPs [85,86]. At a biochemical level, heat acclimation rearranges the level of various metabolites and induces synthesis and/or remobilisation of stress-related compounds. For example, recent results suggest that the galactinol-related pathway may also play a role in the formation of heat acclimation [46]. Polyamines also play a role in adaptation to stresses, as demonstrated under drought, cold stress conditions, and at heavy metal toxicity [87]. Under heat acclimation conditions, a decreased polyamine level was found in wheat plants, accompanied by the increase in 1,3-diaminopropane, a product of the catabolism of polyamines [88]. These results suggest that the positive effect of heat acclimation cannot be characterised by increased polyamine content, but its potential transient elevation or signalling processes associated with the polyamine cycle cannot be excluded from the development of heat tolerance [87]. 

**Figure 2:**
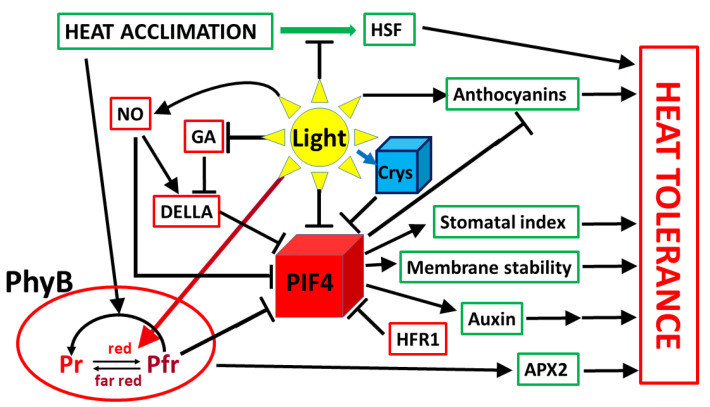
Possible main factors playing a role in light–high-temperature interactions in plants. PIF4 probably plays a central but not exclusive role in the light-mediated acclimation processes, including—among others—regulation of gas exchange, membrane stability, and hormonal regulation. The role of PhyB is controversial. In some processes, it enhances heat tolerance; in others, it reduces heat tolerance. GA: gibberellic acid. For more details, see text.

Light through both photosynthesis and photoreceptors may directly or indirectly affect the development of heat tolerance (Figure 2). However, the role of light in adaptation to elevated temperature has been much less studied than in the case of cold tolerance. Heat stress response is associated with a transient increase in stomatal conductance, which results in elevation of transpiration, the main cooling mechanism in plants. Enhanced transpiration allows the leaves to keep a lower temperature than the environment until protective mechanisms may be activated [83,89,90]. Although heat acclimation at moderate temperatures does not necessarily substantially modify the photosynthetic electron-transport processes and does not always cause photoinhibition, as it usually occurs under cold acclimation conditions, it induces several metabolic changes, which may increase the heat stability of the photosynthetic apparatus [83]. The sensitivity of Rubisco activase enzyme to high temperatures may also determine the heat tolerance of plants [91]. Furthermore, heat acclimated plants are able to activate the regulated heat-dissipation processes, as indicated by the non-photochemical fluorescence-quenching parameters, and they better adjust the stomatal closure at extreme high temperatures than non-acclimated plants [83,92].

Early results indicate that PIF4 plays a dominant central role in acclimation to high temperatures [93]. Acclimation to high temperatures specifically requires PIF4 protein [94,95] and a relatively slight increase in the temperature results in a fast and transient PIF4 expression enhancement [94]. A direct role of the Phy system in heat tolerance has recently been shown in Arabidopsis [72]. Non-heat-acclimated plants grown under low red/far-red conditions, characterised by reduced PhyB activity and increased PIFs abundance, were better able to cope with high temperatures than plants grown at light similar to sunlight. The PhyB and PIF mutant Arabidopsis plants showed high and low heat tolerance, respectively. Reduced expression levels of certain FAD genes found in the PhyB mutant plants suggest that increased membrane stability via altered fatty acid saturation levels may also, at least partly, but probably not solely, explain the PhyB-mediated thermotolerance reduction [72].

PIF4 also plays a central role in integrating hormonal responses and stress signalling at elevated temperatures. Elevating temperature induces auxin synthesis mediated by PIF4. Depending on the temperature, PIF4 is able to bind to the promoters of key auxin biosynthesis genes TAA1 and CYP79B2, and it may activate their expression [7]. Auxin is also able to regulate the expression of numerous genes. Some of them function as negative-feedback regulators of auxin responses. Others, such as small auxin upregulated RNA (SAUR) genes, induce cell elongation, leaf senescence, or cell division [96]. Stress conditions differentially affect the auxin-response-related genes [97]. However, the exact mechanisms are still poorly understood. Auxins may also induce stomatal opening by limiting hydrogen peroxide production in guard cells [98]. PhyB induced by red light may also affect stomatal conductance [99], partially due to elevation of stomata density during development [100]. Transient stomata opening at elevated temperatures is also associated with upregulation of cytokinins and downregulation of ABA [101,102,103]. The decisive role of cytokinins was demonstrated using plants with induced expression of cytokinin biosynthetic gene isopentenyl transferase [104]. So, it can be assumed that PIF4 may also contribute to the long-term heat acclimation. Apart from phytohormones, movement of guard cells induced by high temperature is at least partly regulated by blue-light receptors phototropins, plasma membrane H+-ATPases, multiple members of the 14-3-3 protein family [105], and a downstream target Blue Light Signalling 1 (BLUS1) [106,107]. However, the point in which phototropin-mediated light and temperature signals converge is still unknown.

The level of PIF4 is regulated by several factors. For example, Crys, which play the regulatory role in stress acclimation processes, physically interact with PIF4 and modulate its signal [108]. As a result of this interaction, Cry1 represses auxin biosynthesis in response to elevated temperature [31]. Based on all the mentioned findings, PIF4 may integrate the signals originating from red, as well as blue, light and temperature changes. The role of Crys under different stress conditions has been reviewed; however, better understanding of the Cry role in modulation of temperature responses needs further intensive research [6].

Transition from dark to light may also stimulate the production of nitrogen oxide (NO) and decrease the levels of gibberellins, leading to an increase in DELLA content. DELLA proteins affect transcription factors, including PIFs [109]. PIF4 transcription is also controlled by the circadian clock, with the lowest and highest expression at midnight and at midday, respectively. On the other side, light itself has the opposite effect; it inactivates PIF4 protein during the daytime. Therefore, maximum active PIF4 level can be expected late at night [95]. The transcription factor LONG HYPOCOTYL IN FAR-RED (HFR1) or DELLAs may also repress the action of PIF4 by its sequestering. Inhibition by DELLA can be compensated by GA-mediated proteasomal degradation, leading to enhanced growth [110,111]. 

Just like other stresses, extreme high temperatures, especially in combination with high light, induce oxidative stress [112]. Therefore, the induction of the antioxidant system, which neutralises ROS, can be essential as a protective mechanism. Ascorbate peroxidase 2 (APX2), an important part of the antioxidant system, usually functions under heat stress conditions [113]. One of the recent results suggested that the PhyB-mediated signal may prime the APX2 enzyme, which contributes to the acquired thermotolerance in plants. Exposure of plants to light, especially red light, prior to heat treatment accelerated the thermal induction of the APX2 gene in a PhyB-dependent manner. This priming effect was lacking in PhyB mutants, which could be compromised with exogenous ascorbate, and it was also abolished in Arabidopsis mutants lacking certain heat shock transcription factor HSFA genes. However, PIF mutations did not affect the light-primed thermotolerance, so this PhyB-mediated priming effect is independent of PIF signalling [114]. It must also be mentioned that thermotolerance may involve different mechanisms in etiolated and de-etiolated seedlings. While light-activated PhyB reduced thermotolerance in non-acclimated light-grown and in acclimated etiolated Arabidopsis seedlings, heat acclimation may abolish PhyB effects on thermotolerance of light-grown plants [72]. 

Anthocyanin accumulation may also serve as a marker of various types of stresses to a plant, especially under high light conditions. Anthocyanins provide protection against photo-oxidative damage. Recent results using PIF4 and PIF5 mutants, and PIF4- and PIF5-overexpressing transgenic lines suggested that PIF4 and PIF5 might serve as negative regulators of red-light-induced anthocyanin accumulation. PIFs also influenced the transcription of anthocyanin biosynthesis and regulatory genes in Arabidopsis plants [115].

Although PIF4 probably plays a central role in temperature- and light-mediated processes, other regulatory mechanisms also exist. For example, as it was demonstrated, HSFs are also critical components in heat priming of plants. In contrast to yeast and animals, which usually have only one or a few HSF genes, plants typically contain a much more abundant HSF family [84,116]. An increased expression level of master heat-shock factors HSFA1d and HSFA1e was observed during heat acclimation in the dark, but it was reduced by light [117]. However, the transcription of HSP70 was independent of PIF4, because it was also detected in PIF4 mutants [7].

Heat acclimation usually results in a smaller increase in heat stress tolerance than can be achieved with cold hardening in the induction of freezing tolerance. Accordingly, the role of light is also less known and, in many cases, even controversial in the formation of acquired thermotolerance. However, photoreceptors may significantly influence the level of heat tolerance in plants. 

## 6. Conclusions, Future Questions, and Perspectives

Recent results clearly demonstrate that light significantly affects both low- and high-temperature-dependent responses in plants, which may substantially influence the level of stress injury, or even the survival rate. In some cases, the effect of light can be more important than the genetically determined stress tolerance of the plants. As has been demonstrated, alteration of genes encoding light-regulating components may also modify the stress tolerance of plants. These results might also be used in the future practical plant production. Possible target genes can be the light sensor genes (Phys, Crys, etc.) or their primary interaction partners.

Although the effects of temperature priming have been widely studied, many questions remained to be answered. For example, what is more important, light intensity or quality? Current data indicate that it is both. The effects of photoreceptors on stress tolerance are usually demonstrated on expression levels of certain key genes, such as CBF or COR genes in the case of cold tolerance. However, acclimation to extreme temperature is a complex trait where several mechanisms and crosstalk processes are involved. Results from different photoreceptor mutants show that these plants can be hardened for low temperatures; however, the level of cold tolerance may be different. The crosstalk of temperature signalling with other environmental factors should also be investigated. Furthermore, the adaptation of individual organs to temperature stress differs from that of the whole plant, and recent studies show that light modulation also has an influence on organs which are not directly exposed to the light. A detailed study about the role of, for example, the roots and the crowns is needed. It is still poorly demonstrated, how the particular processes contributing to an enhanced level of stress tolerance are affected by the light spectra. This is particularly important because LED technology is playing an increasing role in crop production, which is the reason why studying the relationship between light and temperature will become more and more important in the near future.

Application of LED technology presents an innovative artificial light source in plant cultivation, as LEDs can provide high fluence and variable spectral composition with relatively low energy consumption and operation temperature, which enable LEDs to be placed close to the leaves, either above the plants or in inter-lighting and intra-canopy irradiation. By now, different kinds of LEDs emitting in the narrow- or wide-bandwidth light spectrum are available, and can be combined according to the desired light composition for plant cultivation in order to ensure appropriate quality and quantity of light for the optimal development adequate to different phases of growth. In addition, LEDs also provide enormous potential in indoor plant cultivation via the possibilities for targeted manipulation of metabolic responses in order to optimise plant productivity and quality. In addition, a better understanding of each light-regulated acclimatisation process may also help breeders to produce more stress-tolerant genotypes.

## Data Availability

No new data were created or analysed in this study. Data sharing is not applicable to this article.
